# Factors associated with empathy among medical students: a cross-sectional study

**DOI:** 10.31744/einstein_journal/2026AO2178

**Published:** 2026-09-10

**Authors:** Anna Carla Travessa Siervo, Alice de Oliveira Hesketh, Pedro Nicolas Brito, Mario Thadeu Leme de Barros, Fernanda Gonçalves Moreira, Thiago Marques Fidalgo, Ângela Tavares Paes, Marcelo Feijó Mello

**Affiliations:** 1 Faculdade Israelita de Ciências da Saúde Albert Einstein Hospital Israelita Albert Einstein São Paulo SP Brazil Faculdade Israelita de Ciências da Saúde Albert Einstein, Hospital Israelita Albert Einstein, São Paulo, SP, Brazil.; 2 Escola Paulista de Medicina Universidade Federal de São Paulo São Paulo SP Brazil Escola Paulista de Medicina, Universidade Federal de São Paulo, São Paulo, SP, Brazil.

**Keywords:** Empathy, Student, medical, Mental health, Anxiety, Spirituality, Religion, Risk factors, Surveys and questionnaires

## Abstract

**Objective:**

To examine factors associated with empathy among medical students, focusing on sociodemographic, academic, and personal characteristics.

**Methods:**

A cross-sectional study was conducted with 580 medical students from all academic years. Empathy was assessed using the Interpersonal Reactivity Index. Independent variables included sociodemographic data (age, gender, socioeconomic status, and religion), academic characteristics (year of study, career intention, and extracurricular activities), and personal factors (mental health, self-perceived empathy, and satisfaction with life domains). Descriptive analyses and multivariate linear regression models were conducted.

**Results:**

The mean empathy score was 99.6 (SD=11.9), indicating relatively high empathy levels. Among the four subscales, Personal Distress showed the greatest variability. Multivariate regression revealed that higher empathy scores were independently associated with females (β=+6.1, p<0.001), religion or spirituality (β=+3.1 to +3.8, p<0.05), preference for clinical specialties (β=+4.2 to +9.3, p<0.001), and anxiety symptoms (β=+4.0, p<0.001). Interaction analyses indicated that anxiety had a stronger positive effect on empathy among male students and those with religiosity or spirituality. Variables such as institution, year of study, age at admission, financial factors, depressive symptoms, substance use, and satisfaction with life domains were not significantly associated.

**Conclusion:**

Empathy among medical students is influenced by sociodemographic, academic, and personal factors. Female gender, religion/spirituality, clinical specialty interest, and anxiety symptoms were positively associated with empathy. These findings support integrating structured empathy training into medical curricula, considering psychological support, reflective practice, and cultural–spiritual dimensions to preserve and enhance empathy throughout medical education.

## Highlights

■ Female gender and spirituality correlate with higher empathy levels.

■ Anxiety enhances empathy, with a stronger effect in male and religious students.

■ Lowest empathy observed in non-religious males seeking surgical specialties.

■ Age, income, academic year, and depression show no significant association with empathy.

## In Brief

Siervo et al. investigate the factors associated with empathy among medical students. Their findings reveal that female gender, religion/spirituality, interest in clinical specialties, and anxiety symptoms are positively associated with empathy. Interaction analyses indicate that anxiety has a stronger positive effect on empathy among male students and those who are religious or spiritual.

## Graphical abstract

**Figure:**
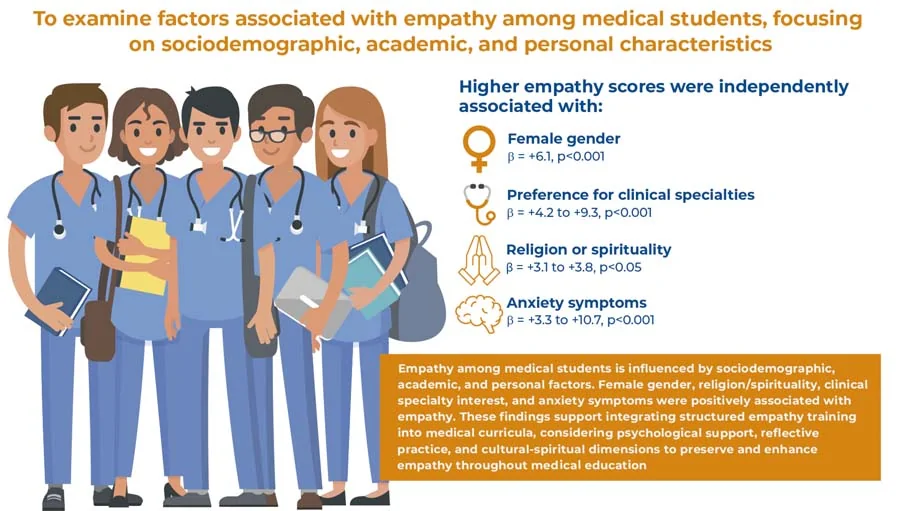

## INTRODUCTION

Empathic communication is fundamental to high-quality medical practice, with direct implications for treatment adherence, clinical outcomes, and patient satisfaction.^(1-6)^In a study with diabetes patients, Hojat et al.^(7)^demonstrated that glycated hemoglobin (HbA1c) control (<7.0%) and low-density lipoprotein cholesterol (LDL-C) levels (<100mg/dL) were significantly higher among patients treated by highly empathic physicians compared to those with low empathy (56% *versus* 40% and 59% *versus* 44%, respectively; p<0.01). In another study, the same author reported a strong correlation (r=0.93) between patient satisfaction scores and physician empathy. ^(7)^ These findings underscore the importance of a comprehensive understanding of empathy, its various dimensions, and its manifestations in clinical interactions.

Davis^(8,9)^ defined empathy as a multidimensional construct encompassing cognitive and emotional components, responsible for recognizing emotions and reacting to these stimuli, respectively.^(8,9)^ Suchman et al.^(1)^observed that during medical consultations, patients rarely verbalized their emotions directly and spontaneously, offering indirect cues. Therefore, physicians must rely on empathy to identify such emotions and enhance the quality of the clinical interview.

The development of medical skills begins in undergraduate training. Empathy is recognized as an essential competency in the Brazilian National Curricular Guidelines for medical education.^(10)^ However, several international studies have raised concerns about a decline in empathy throughout medical training.^(11-14)^ Newton et al.^(15)^suggested that a reduction in emotional responsiveness occurred during medical school, possibly as a reaction to the academically demanding environment and repeated exposure to suffering. Therefore, it is critical to investigate the factors associated with medical students’ empathic capacity.^(15-20)^

In a systematic review of 18 studies, Neumann et al.^(15)^ examined associations between empathy and variables such as gender, age, mental health, curriculum, and specialty preferences. However, they emphasized that only a limited set of possible variables had been studied. In Brazil, research explored factors similar to those internationally studied.^(21-23)^ However, it is essential to consider sociocultural, interpersonal, and academic particularities that may influence the development of empathy among Brazilian students. Hence, the present study examines sociodemographic, academic, and mental health factors associated with empathy among Brazilian medical students.

## OBJECTIVE

This investigation provided a comprehensive and contextualized understanding of empathy throughout medical training, contributing to the development of a humanized, practical, and patient-centered clinical practice.

## METHODS

### Study design

This is a cross-sectional study involving undergraduate medical students from the 1st to the 6th year at two institutions in São Paulo, Brazil: the *Faculdade Israelita de Ciências da Saúde Albert Einstein* (FICSAE/Einstein), a private institution with an active learning methodology, including team-based learning, and the *Escola Paulista de Medicina da Universidade Federal de São Paulo* (EPM/UNIFESP), a public institution with a traditional teaching model. Data was collected between March and April 2024. Information was obtained using a self-administered questionnaire developed on the REDCap (Research Electronic Data Capture) platform.^(24)^ In addition to empathy, questions included sociodemographic, economic, academic, and mental health characteristics, and satisfaction with academic routine. This study was approved by the Research Ethics Committee of *Hospital Israelita Albert Einstein* (HIAE), CAAE: 75961323.8.1001.0071; # 6,676,128. All the participants signed an informed consent form, as required by the ethical guidelines of CNS Resolution 466/2012. The authors declare that they have no conflicts of interest.

### Data collection

All undergraduate medical students from the 1st to the 6th year at FICSAE/Einstein and EPM/UNIFESP were invited to participate.

The invitation was disseminated through the WhatsApp messaging application using announcement groups specific to each class. Every class in both institutions maintained such groups for communication and information sharing. Each group had student representatives responsible for forwarding messages to their peers. The research team obtained the representatives’ contact information through institutional channels, and they distributed the study invitation to their respective classes. The invitation included a link to an electronic form containing the project description, the informed consent form, and the questionnaire. Additionally, in-class announcements were made, with QR codes provided to direct students to the form and consent document.

### Questionnaire

The questionnaire consisted of three sections (Table 1S, Supplementary Material).

1. Sociodemographic and academic variables: Gender, date of birth, religion or spirituality, per capita income, financial support for studies, employment, semester and year of the course, age at entry into medical school, institution, and preference for clinical or surgical specialties.
2. Mental health, lifestyle, and personal perceptions: Screening for generalized anxiety disorder (Generalized Anxiety Disorder 2-item scale, GAD-2),^(25)^ consisting of two questions. These were scored on a Likert scale from 0 to 3. The total score consisted of 0–6, with ≥3 indicating a positive screen. Screening for depressive symptoms (Patient Health Questionnaire-2, PHQ-2)^(26)^ were scored similarly. Screening for substance use problems, Two-item Conjoint Screen (TICS),^(27)^ with two yes/no questions, where a single positive response indicated risk. Additional items included self-reported use of psychiatric medications (including antidepressants, anxiolytics, and antipsychotics), utilization of psychology/psychiatry services, and Likert-type scales assessing self-perceived empathy, motivation for choosing a career in medicine, social support, overall satisfaction with the academic routine, and frequency of volunteer activities.
3. Empathy assessment: described below.

### Empathy measurement

Empathy was measured using the Interpersonal Reactivity Index (IRI) developed by Davis^(9)^ and validated in Portuguese.^(28)^ This instrument consisted of 26 statements describing behaviors, feelings, and characteristics related to dispositional empathy. It reflected stable, trait-like individual differences in cognitive and affective responsiveness to others’ experiences, rather than empathy expressed in response to specific situations or clinical contexts.

Beyond the total score, it evaluated four subscales: perspective taking (PT), fantasy (FS), empathic concern (EC), and personal distress (PD).

- Perspective taking: Reflected social functioning and non-egocentric behavior, including the ability to adopt others’ viewpoints, consider different opinions, and anticipate others’ reactions.
- Fantasy: Related to emotional involvement with fictional scenarios, such as imagining oneself in the place of characters in books, films, or stories.
- Empathic concern: Reflected emotional responsiveness toward others, such as feelings of sympathy, compassion, and concern for people in adverse situations.
- Personal distress: Reflected self-oriented emotional reactivity, including anxiety, tension, and discomfort in response to others’ distress.

Each subscale included six (PT, PD) or seven (FS, EC) items, rated from 1 (“does not describe me well”) to 5 (“describes me very well”). Total scores ranged from 26 to 130, with higher scores indicating higher empathy. One item was reverse-scored due to translation adaptations.^(28)^

### Statistical analysis

The statistical analysis characterized the sample and identified factors associated with students’ empathic capacity. Independent variables included sociodemographic characteristics (gender, age, institution, year of study, income, financial aid, employment), aspects related to the choice of medicine and extracurricular activities (motivation for choosing the course, volunteer work, preference for clinical or surgical specialties), mental health profile PHQ-2 for depressive symptoms and anhedonia, GAD-2 for anxiety symptoms, TICS for substance use, and use of mental health services), social support (support network, satisfaction with social circle), and general satisfaction (academic routine, personal life). The outcome was the total IRI empathy score and its subscales (PT, FS, EC, and PD).

Categorical variables were summarized by absolute and relative frequencies; quantitative variables were expressed as means, medians, standard deviations (SDs), minimum and maximum values, or categorized as appropriate. Group comparisons of IRI dimensions scores were performed using analysis of variance (ANOVA) or the Kruskal–Wallis test, depending on distribution. To identify factors associated with empathy, linear regression models were applied, with the IRI total score as the dependent variable. Model assumptions, including normality and homoscedasticity, were verified through residual analyses and diagnostic plots. Univariate analysis was performed to examine the isolated influence of each factor on empathy. Variables with p<0.15 were subsequently included in multiple regression models, with p<0.05 required for retention in the final model. Multicollinearity and interactions between variables were tested as well. Statistical analyses were conducted using RStudio (RStudio PBC, Boston, MA, USA) and JAMOVI software.

### Data storage

All research data and documents were stored on the REDCap platform at Einstein. Data were anonymized by removing participant identifiers and replacing them with codes accessible only to the principal investigators. Access to REDCap data was restricted to the leading researchers through password authentication. The data were stored on an internal secure server at HIAE, managed by the hospital’s Information Technology department. The server utilized mirrored hard drives to ensure data backup in the event of hardware failure, guaranteeing data integrity and restricting access. Despite anonymization, the dataset contains sensitive information and unrestricted public sharing could risk re-identification. Therefore, the data are not publicly available. However, it may be provided by the corresponding author upon reasonable request.

## RESULTS

### Sample characteristics

The total number of students enrolled in both institutions was 1,446 (726 at EPM/UNIFESP and 720 at FICSAE/Einstein). The initial sample consisted of 722 (49.9%) students. During data cleaning, 142 participants were excluded due to incomplete questionnaires, resulting in a final sample of 580 (80.3% of the respondents).

Of the total participants, 57.1% were from FICSAE and 42.9% from EPM. As shown in table 1, the highest response rates were observed in the 1st and 2nd years, representing 54% of the sample (Figure 1). The sample was predominantly female (62%), aged between 19 and 25 years (65%), and reported some form of religion or spirituality (83%). The economic profile was assessed by per capita income, with 50% of students having a per capita income above BRL 5,000. Most participants did not work (80%), and 22% reported receiving financial aid (scholarships, student loans, or housing, transportation, and food assistance). Sociodemographic differences were noted between the institutions. *Escola Paulista de Medicina da Universidade Federal de São Paulo* students entered medical school slightly older, included a higher proportion of males, and had lower household income.

**Table 1:** Sociodemographic and academic data

Characteristic	n (%)
Gender	
Female	361 (62.2)
Male	216 (37.2)
Non-binary	3 (0.5)
Medical School	
FICSAE/Einstein	331 (57.1)
EPM/UNIFESP	249 (42.9)
Year of study	
1st	163 (28.1)
2nd	151 (26.0)
3rd	86 (14.8)
4th	61 (10.5)
5th	60 (10.3)
6th	59 (10.2)
Age at enrollment (in years)	
≤18	134 (23.2)
19–24	375 (65.0)
≥25	68 (11.8)
Religion/Spirituality	
Practicing religious	171 (29.5)
Non-practicing religious	171 (29.5)
Spiritual but not religious	138 (23.8)
Neither spiritual nor religious	100 (17.2)
Religion	
Catholic	194 (33.4)
Evangelical	79 (13.6)
Spiritist	30 (5.2)
Umbanda, Candomblé, or other Afro-Brazilian religions	14 (2.4)
Jewish	12 (2.1)
Other	12 (2.1)
No response	1 (0.2)
No religion	238 (41)
Income (in BRL)	
Less than 1000 per capita	27 (4.7)
1001–3000	164 (28.3)
3001–5000	98 (16.9)
More than 5000 per capita	291 (50.2)
Reason for Choosing Medicine	
Interest in patient care	125 (21.6)
Academic/scientific interest	137 (23.6)
Humanitarian interest	184 (31.7)
Social prestige	5 (0.9)
Financial stability/monetary benefit	28 (4.8)
Scope of future professional practice	55 (9.5)
Family imposition/request	3 (0.5)
Only career perspective	29 (5)
No interest in medicine	2 (0.3)
Other	12 (2.1)
Preference for Clinical or Surgical Specialty	
Strong preference for surgical specialty	60 (10.3)
Preference for surgical	155 (26.7)
No preference/Equal interest	107 (18.4)
Preference for clinical	175 (30.2)
Strong preference for clinical specialty	83 (14.3)
Total	580 (100.0)

FICSAE: Faculdade Israelita de Ciências da Saúde Albert Einstein; EPM/UNIFESP: Escola Paulista de Medicina da Universidade Federal de São Paulo.

**Figure 1:**
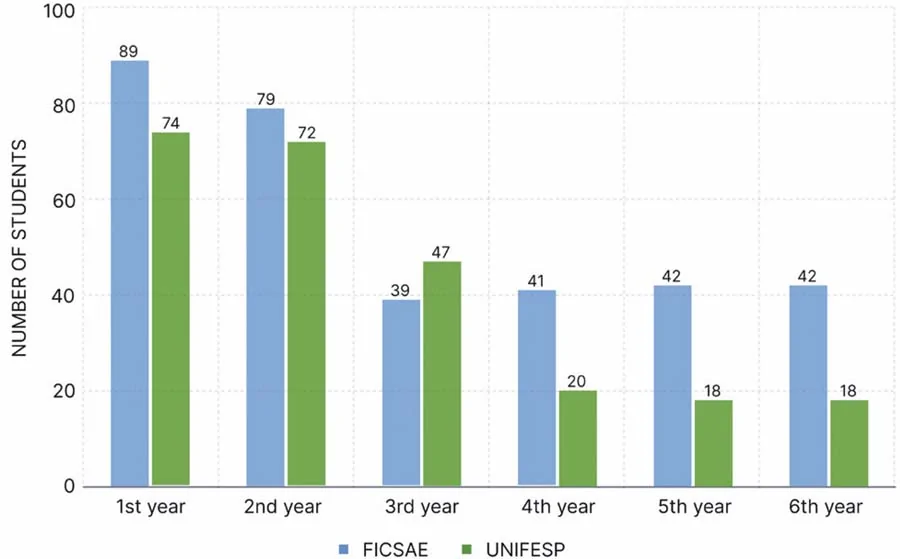
Distribution of respondents by year of study and medical school

Regarding motivation for studying medicine, 31% reported humanitarian reasons, 23% cited scientific interest, and 21% expressed an interest in assistance-related fields. Volunteer work was reported as frequent or very frequent in 20% of the students. The preference for clinical versus surgical specialties was balanced, with a slight tendency toward clinical fields (44%) compared to surgical fields (37%), and an equal number of interested students (18%). This distribution was similar across genders (p=0.15) and institutions (p=0.20).

As shown in table 2, 20.9% screened positive on the PHQ-2 for depressive mood and anhedonia in the previous two weeks, 37.1% screened positive on the GAD-2 for generalized anxiety disorder, and 33.8% screened positive on the TICS for substance use problems in the previous year. Notably, 50.2% reported having used mental health resources in the past six months, among whom 49.8% screened positive for anxiety (GAD-2) and 29% for depression (PHQ-2).

**Table 2:** Mental health data and social support

Characteristic	n (%)
PHQ 2 – Cut-off	
Below cut-off (0–2)	459 (79.1)
Above cut-off (3–6)	121 (20.9)
GAD 2 – Cut-off	
Below cut-off (0–2)	365 (62.9)
Above cut-off (3–6)	215 (37.1)
TICS	
Negative	384 (66.2)
Positive	196 (33.8)
Use of Mental Health Services and Medications	
Used one or more resources	291 (50.2)
Psychologist	212 (36.6)
Psychiatrist	123 (21.2)
Antidepressant	124 (21.4)
Anxiolytic	112 (19.3)
Antipsychotic	21 (3.6)
Did not use any resources	289 (49.8)
Number of People in Support Network	
None	7 (1.2)
1–2	67 (11.6)
3–5	267 (46)
5–10	192 (33.1)
>10	47 (8.1)
Satisfaction with Social Support	
Not at all satisfied	5 (0.9)
Slightly satisfied	29 (5)
Neutral	29 (5)
Moderately satisfied	172 (29.7)
Very satisfied	345 (59.5)
Satisfaction with Medical Routine	
Not at all satisfied	7 (1.2)
Slightly satisfied	61 (10.5)
Neutral	78 (13.4)
Moderately satisfied	271 (46.7)
Very satisfied	163 (28.1)
Satisfaction with Personal Life	
Not at all satisfied	11 (1.9)
Slightly satisfied	80 (13.8)
Neutral	64 (11)
Moderately satisfied	264 (45.5)
Very satisfied	161 (27.8)
Total	580 (100.0)

PHQ-2: Patient Health Questionnaire-2; GAD 2: Generalized Anxiety Disorder Scale-2; TICS: Two-item conjoint screen.

When asked about the number of people they could contact in times of difficulty, 46% reported having between 3 and 5 contacts, and 33.1% reported having between 5 and 10 contacts. Overall, students reported high satisfaction with social support, academic routine, and personal life.

### Empathy profile

The mean total empathy score on the IRI was 99.6 (median=100), with a SD=11.87, ranging from 51 to 130. When transformed to a 0–100 scale, this corresponded to a mean of 70.8. Figure 2 shows the distribution of total and subscale scores standardized to a 0–100 scale. PT, FS, and EC displayed higher score concentrations, while PD showed greater variability.

**Figure 2:**
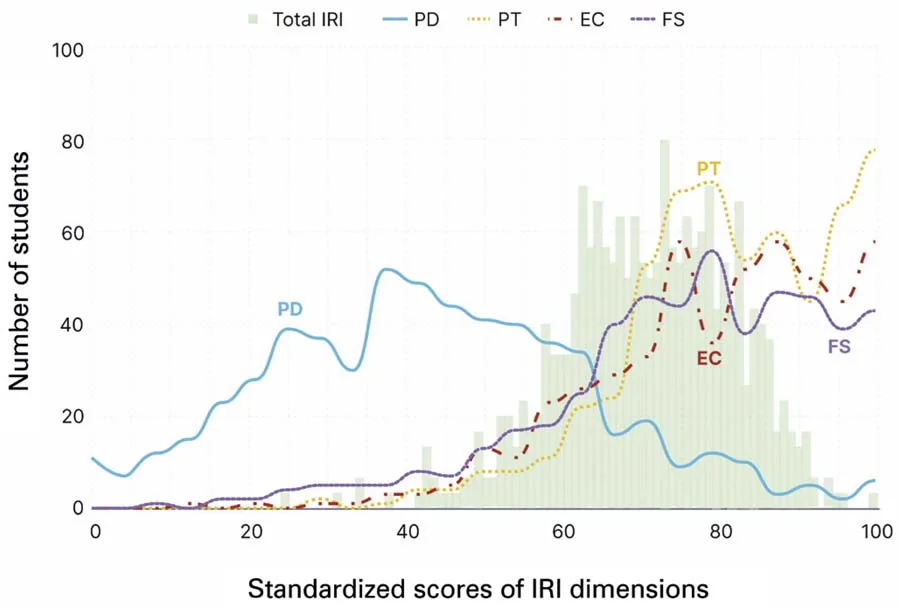
Distribution of scores on the IRI dimensions on standardized 0–100 scales

PT: perspective taking; FS: fantasy; EC: empathic concern; PD: personal distress.

### Factors associated with empathy

In univariate analyses, variables associated with dispositional empathy at p<0.15 included gender, religion/spirituality, preference for clinical or surgical specialties, self-perceived empathy, social support networks, engagement in volunteer activities, anxiety symptoms, and use of mental health services (Table 2S, Supplementary Material). No statistically significant variation in empathy scores was observed throughout the medical course. The remaining variables (including institution, age at enrollment, type of religion, satisfaction with social life, satisfaction with academic routine, satisfaction with personal life, PHQ-2 depression screen, TICS substance use, income, financial aid, or employment) did not meet the p<0.15 criterion for inclusion in the multivariable model.

In multivariate analysis, the following variables remained significantly associated with higher empathy: female gender, preference for clinical specialties, anxiety symptoms, and religion/spirituality (Table 3). Collinearity was identified between self-perception of empathy and religion/spirituality; the latter was retained in the final model due to greater objectivity.

**Table 3:** Linear regression model results (multivariate analysis)

Variables	Multivariate Analysis		
	Beta	Standard error	p value
Gender			
Female	91.73	1.8	
Male	-6.12	0.957	<0.001
Non-binary	+7.11	6.371	0.265
Religion/Spirituality			
Neither spiritual nor religious	91.73	1.8	
Practicing religious	+3.05	3.05	0.029
Non-practicing religious	+3.79	3.79	0.006
Spiritual but not religious	+3.11	3.11	0.033
Preference for Clinical or Surgical Specialty			
Strong preference for surgical specialty	91.73	1.8	
Preference for surgical	+4.2	1.661	0.012
No preference/Equal interest	+6.9	1.769	<0.001
Preference for clinical	+7.06	1.639	<0.001
Strong preference for clinical specialty	+9.33	1.851	<0.001
GAD 2 (Cut-off)			
Below cut-off	91.73	1.8	
Above cut-off	+3.99	0.944	<0.001

GAD 2: Generalized Anxiety Disorder Scale-2.

On average, female students scored 6.1 points higher than males (95%CI=4.24–8.0, p<0.001). Positive anxiety screening (GAD-2 ≥3) was associated with a 4-point increase (p<0.001). Considering the GAD-2 scale (0–6), maximum scores corresponded to an average increase of 10.7 points compared to minimum scores (p<0.001) (Figure 3 and Table 3S, Supplementary Material). Religion/spirituality was associated with higher empathy. Compared to students without religion/spirituality, empathy scores were on average 3.05 (p=0.02), 3.79 (p=0.006), and 3.11 (p=0.03) points higher for practicing religious, non-practicing religious, and spiritual students, respectively, with no significant differences between these subgroups. A preference for clinical specialties was linked to higher empathy as well, with increments ranging from 4.2 to 9.3 points compared to an exclusive preference for surgery (p<0.05).

**Figure 3:**
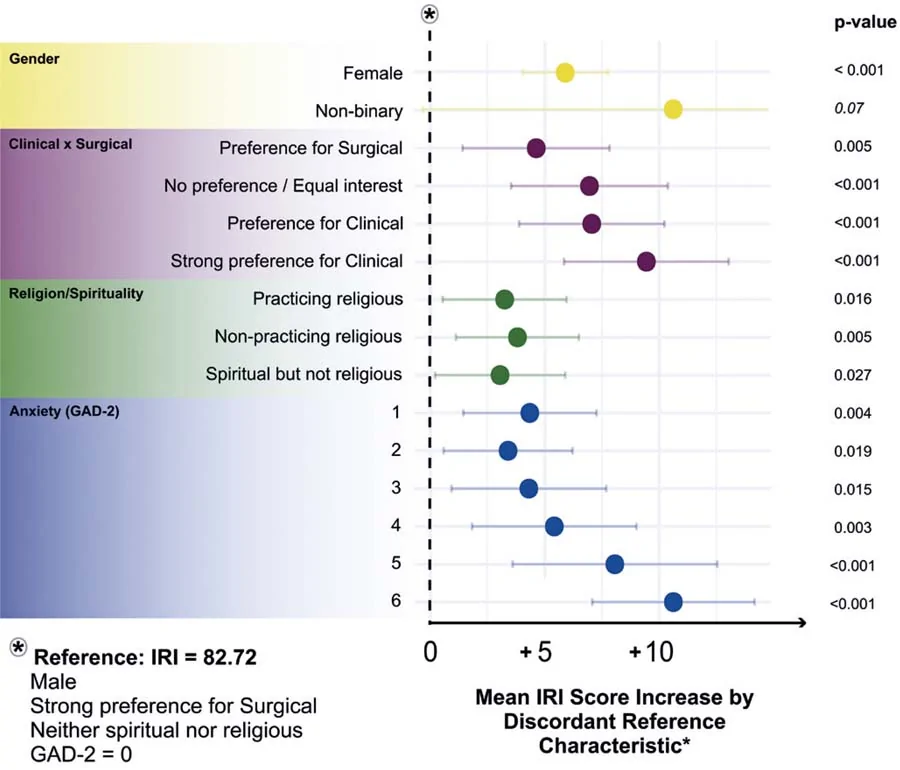
Regression model coefficients based on GAD-2 across all scores

Subscale analyses revealed that FS, EC, and PD drove gender and anxiety effects, while PT remained stable. Religion/spirituality differences were observed only in EC, whereas clinical versus surgical preference influenced all subscales (Table 4). Pearson correlation showed positive associations between anxiety (GAD-2) and FS (r=0.185), EC (r=0.161), and PD (r=0.252). Interaction analyses revealed significant effects between gender and anxiety (p=0.003). Anxiety increased empathy by 7 points in males versus 2.5 points in females (Figure 4A). Due to the limited number of non-binary participants (n=3), they were excluded from the interaction analysis. Religion/spirituality and anxiety interacted (p<0.05): students without religion/spirituality did not show increased empathy with anxiety, unlike other groups (Figure 4B). No other significant interactions were identified.

**Table 4:** Comparisons of Interpersonal Reactivity Index dimension scores for each variable in the multivariate model

	Total IRI (95%CI)	PD (95%CI)	PT (95%CI)	EC (95%CI)	FS (95%CI)
Gender					
Female	102.1 (101–103.2)	16.9 (16.4–17.4)	25.8 (25.5–26.1)	30.4 (30–30.8)	29 (28.6–29.5)
Male	95.2 (93.5–96.9)	15.6 (14.9–16.3)	25.3 (24.9–25.8)	27.7 (27.1–28.3)	26.6 (25.9–27.3)
p-value	<0.001**	0.004**	0.106	<0.001**	<0.001**
GAD-2					
Below cut-off	97.8 (96.6–99.1)	15.6 (15.1–16.1)	25.6 (25.3–25.9)	29.1 (28.6–29.5)	27.6 (27.1–28.1)
Above cut-off	102.4 (100.8–103.9)	17.8 (17.1–18.5)	25.7 (25.2–26.1)	29.9 (29.3–30.4)	29.1 (28.4–29.7)
p-value	<0.001**	<0.001**	0.781	0.033**	<0.001**
Preference for Clinical or Surgical Specialty					
Strong preference for surgical specialty	92.8 (89.3–96.2)	14.2 (12.9–15.6)	24.4 (23.4–25.5)	28 (26.8–29.3)	26 (24.5–27.6)
Preference for surgical	97.9 (96–99.7)	15.9 (15.2–16.6)	25.5 (25.1–26)	29 (28.3–29.7)	27.4 (26.5–28.3)
No preference/Equal interest	101.3 (99–103.5)	16.9 (15.9–17.9)	25.9 (25.2–26.5)	29.9 (29–30.7)	28.6 (27.7–29.6)
Preference for clinical	100.8 (99.3–102.4)	16.9 (16.2–17.6)	25.6 (25.2–26.1)	29.5 (28.9–30.1)	28.8 (28.1–29.4)
Strong preference for clinical specialty	102.5 (99.9–105.1)	17.3 (16.1–18.5)	26.3 (25.5–27)	30.1 (29.1–31.1)	28.9 (27.8–29.9)
p-value	<0.001**	<0.001**	0.021**	0.024**	<0.001**
Religion/Spirituality					
Practicing religious	100.3 (98.6–102)	16.8 (16–17.5)	25.5 (25–26)	29.9 (29.3–30.5)	28.1 (27.4–28.9)
Non-practicing religious	100.2 (98.6–101.9)	16.6 (15.8–17.4)	26 (25.5–26.5)	29.5 (28.9–30.1)	28.2 (27.5–28.9)
Spiritual but not religious	100.5 (98.6–102.4)	16.3 (15.6–17.1)	25.7 (25.1–26.2)	29.7 (29–30.5)	28.8 (28–29.6)
Neither spiritual nor religious	95.5 (92.8–98.2)	15.5 (14.5–16.6)	25.2 (24.6–25.9)	27.7 (26.7–28.6)	27.1 (25.9–28.3)
p-value	0.003**	0.241	0.298	<0.001**	0.088

**p<0.05.

PT: perspective taking; FS: fantasy; EC: empathic concern; PD: personal distress; 95%CI: 95% confidence interval.

**Figure 4:**
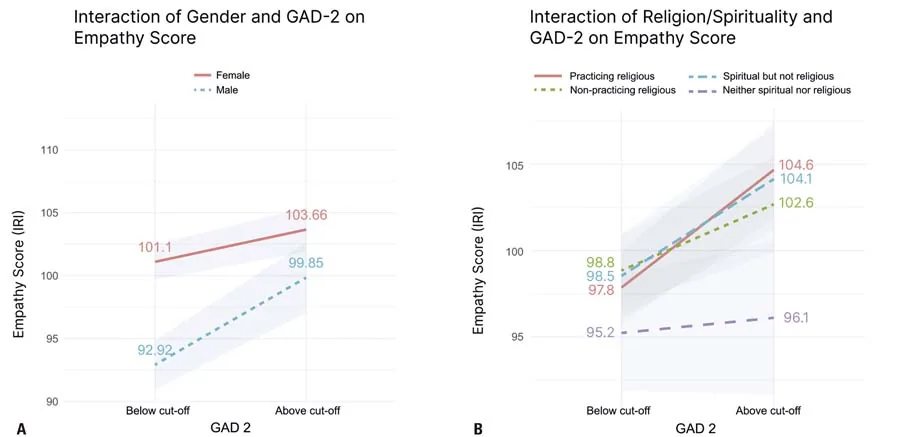
Interaction plots of the Generalized Anxiety Disorder Scale-2 with gender and religion/spirituality

## DISCUSSION

This study identified that empathy among medical students was associated with gender, preference for clinical or surgical specialties, religion/spirituality, and anxiety symptoms measured by GAD-2. Female students and those interested in clinical specialties demonstrated higher empathy, which was consistent with multiple studies.^(8,9,11,14,17-22)^ Furthermore, having any form of religion or spirituality was associated with higher empathy compared to students without such beliefs, with no difference between specific religions. Literature on this subject remains mixed. A 2023 Brazilian study demonstrated a significant association between higher spirituality levels and increased empathy, as measured by the IRI (p<0.001).^(29)^ Thomas reported similar findings in the United States,^(30)^ who observed higher empathy among physicians with intense spirituality or a sense of purpose.

In contrast, Santos et al.^(21)^ and Damiano et al. ^(31)^ did not find such associations using self-reports of religious belief and the WHOQOL-Spirituality, Religiousness, and Personal Beliefs scale, respectively. Notably, in this study, the variation in empathy based on religion/spirituality was mainly driven by EC, which captured affective responses, such as sympathy and compassion. Furthermore, Pargament argued that spirituality fostered a sense of transcendence and connection with others, which promoted empathic and compassionate behaviors.^(32)^

Students with positive GAD-2 screening demonstrated higher empathy, with mean differences of up to 10 points on the IRI when comparing maximum versus minimum anxiety scores. Although psychiatric medication use and access to mental health services were common, they were not significantly associated with empathy after adjustment. A meta-analysis by Nair et al.^(33)^ found a weak yet significant correlation between anxiety and empathy (r=0.08), which was stronger for affective empathy (r=0.16) than for cognitive empathy (r=-0.03). Geographical region moderated these effects, with collectivist cultures showing stronger associations. In this study, PD was moderately correlated with anxiety (r=0.252), slightly higher than values reported in the meta-analysis. One possible explanation was that anxiety heightened sensitivity to others’ emotions and increased interpersonal awareness, which manifested as higher empathy. Cha et al.,^(34)^ studying cancer patients, reported that anxiety negatively affected spirituality and quality of life. However, it was positively associated with interpersonal coping, suggesting a similar mechanism. Given the cross-sectional design, causality could not be inferred. Hence, it remained unclear whether anxiety promoted empathy or whether higher empathy predisposed to anxiety.

Interaction analysis revealed that anxiety had a more substantial impact on empathy among male students than females, raising questions about emotional regulation, social expectations, and biological differences between genders. Anxiety interacted differently depending on the presence of religion/spirituality. Among students without such beliefs, anxiety was not associated with higher empathy, unlike in other groups. Shabani et al^(35)^ demonstrated that spirituality and resilience mediated the relationship between anxiety and life satisfaction, suggesting that spirituality acted as a coping mechanism that channeled anxiety into more empathic responses.

Some variables were not significantly associated with overall empathy scores. Year of study did not correlate with empathy, although the literature remains divided. While some studies suggested a progressive decline throughout medical training ^(11)^ others, including extensive reviews of international data, did not confirm this pattern. ^(19-21)^ Social support, though critical for student well-being, was not significantly related to empathy in this sample, contradicting Capdevila-Gaudens, who identified such an association.^(17)^

### Limitations

This study has certain limitations. The cross-sectional nature of the study limited the ability to infer causal relationships among the observed associations. The reliance on self-administered questionnaires may have led to response bias, as participants may have answered in ways that they perceived as socially acceptable. Importantly, recruitment through text-based invitations raised the possibility of selection bias, where students with higher empathy levels or greater interest in psychosocial topics were more likely to respond to the survey. This resulted in an overrepresentation of empathetic individuals and the overall empathy levels in the sample. Participation from students in the final years was lower, potentially reducing the power to detect differences across stages of the course.

## CONCLUSION

This study found that higher dispositional empathy among medical students was associated with female gender, preference for clinical specialties, presence of anxiety symptoms, and religion/spirituality. Interactions indicated that anxiety affected empathy differently depending on gender and religion/spirituality. These findings highlight the importance of curricular strategies in promoting empathy, strengthening mental health support, and acknowledging personal dimensions, such as spirituality. Future longitudinal studies must clarify the relationship between empathy, mental health, and individual factors throughout medical training. A longitudinal follow-up of this cohort is currently underway, which will allow for a more robust examination of these dynamics over time.

## SUPPLEMENTARY MATERIAL

Supplementary material
