# Supplementary material for: Factors associated with empathy among medical students: a cross-sectional study
Source: Einstein (Sao Paulo). 2026 Sep 10;24:eAO2178. doi: 10.31744/einstein_journal/2026AO2178 (PMC13682782; doi:10.31744/einstein_journal/2026AO2178)
Supplement: Supplementary file 1 [file 2317-6385-eins-24-eAO2178-suppl01.pdf]

SUPPLEMENTARY MATERIAL

Factors associated with empathy among medical students: a cross-sectional study

Anna Carla Travessa Siervo, Alice de Oliveira Hesketh, Pedro Nicolas Brito, Mario Thadeu Leme de Barros Filho, Fernanda Gonçalves Moreira, Thiago Marques Fidalgo, Ângela Tavares Paes, Marcelo Feijó Mello

DOI: 10.31744/einstein_journal/2026AO2178

Table 1S. Self-administered questionnaire filled out by the participants

**Informed Consent Form**
• I agree to participate in the study
• I do not agree to participate in the study
**Name**
**E-mail**
**Phone number**
**Sex**
• Female
• Male
• Non binary
• Other
**Date of birth**
**College**
• FICSAE
• UNIFESP
**Which semester/year of medical school are you currently attending?**
• 1st semester/1st year
• 2nd semester/1st year
• 3rd semester/2nd year
• 4th semester/2nd year
• 5th semester/3rd year
• 6th semester/3rd year
• 7th semester/4th year
• 8th semester/4th year
• 9th semester/5th year
• 10th semester/5th year
• 11th semester/6th year
• 12th semester/6th year
**At what age did you start medical school?**
**What is your relationship with religion?**
• I have a religion and am a practicing member
• I have a religion but am not practicing
• I do not have a religion but have a spirituality
• I have neither religion nor spirituality
**If you have a religion, which one?**
• Catholic
• Evangelical
• Spiritist
• Umbanda, Candomblé, or other Afro-Brazilian religions
• Jewish
• Other

continue...

...Continuation

Table 1S. Self-administered questionnaire filled out by the participants

"I consider myself very empathetic"

1 – 5 Likert scale

- 1=Strongly disagree
- 2=Partially disagree
- 3=Neutral/Unable to evaluate
- 4=Partially agree
- 5=Strongly agree

What is your personal interest/professional inclination between clinical or surgical areas?

1 – 5 Likert scale

- 1=Extreme preference for surgery
- 2=Preference for surgery
- 3=No preference/Equal interest
- 4=Preference for clinical
- 5=Extreme preference for clinical

Select your primary motivation for choosing a career in medicine (before entering medical school):

- Interest in patient care
- Academic/Scientific interest
- Humanitarian interest
- Social prestige
- Financial stability/Monetary benefit
- Scope of future professional practice
- Family imposition or request
- Only career option
- No interest in medicine
- Other

Social Support and Satisfaction

How many people could you rely on if you were facing serious personal issues?

- None
- 1–2
- 3–5
- 6–10
- 10

At this moment, how satisfied are you with the support you receive from your social circle?

- Not at all satisfied
- Slightly satisfied
- Neutral
- Moderately satisfied
- Very satisfied

At this moment, how satisfied are you with your medical practice/academic routine?

- Not at all satisfied
- Slightly satisfied
- Neutral
- Moderately satisfied
- Very satisfied

At this moment, how satisfied are you with your personal life?

- Not at all satisfied
- Slightly satisfied
- Neutral
- Moderately satisfied
- Very satisfied

continue...

...Continuation

Table 1S. Self-administered questionnaire filled out by the participants

In the last 6 months, have you engaged in charity, philanthropy, or volunteer activities with a social focus?

1 – 5 Likert scale

- 1=Never
- 2=Rarely
- 3=Occasionally
- 4=Frequently
- 5=Very frequently

Empathy Instrument

INSTRUCTIONS: Below are descriptive behavioral statements. For each statement, select the response that best reflects **YOUR** behavior. Be honest and answer carefully. Mark **only one option** per item. Do not leave any item blank.

1 – 5 Likert scale

- 1=Does not describe me well
- 5=Describes me very well

Examples:

- I easily take the perspective of a character in a movie.
- After watching a play or a movie, I feel involved with the characters.
- I usually get emotionally involved with movies and/or books.
- I feel the emotions of a movie character as if they were my own.
- I am neutral when watching movies.
- I often imagine things that could happen to me.
- When I see an interesting story, I imagine how I would feel if it happened to me.
- I care about people who do not have a good quality of life.
- I feel compassion when someone is treated unfairly.
- I am disturbed by bad things that happen to others.
- I am moved by other people's problems.
- When I see someone being taken advantage of, I feel the need to protect them.
- I often get emotional about things happening to others.
- I consider myself a "soft-hearted" person (very sensitive).
- I usually get nervous when seeing injured people.
- I feel apprehensive in emergencies.
- I feel tense in highly emotional situations.
- I tend to lose control during emergencies.
- I feel helpless in an emotional situation.
- I lose control when I see someone in great need of help.
- I try to understand my friends by imagining how they see things.
- I listen to others' arguments even when I am confident in my own opinion.
- Before making a decision, I try to evaluate all perspectives.
- I try to understand other people's arguments.
- I imagine how people feel when I criticize them.
- I put myself in others' shoes if I care about them.

PHQ-2 Screening

In the past 2 weeks, how often have you been bothered by the following symptoms?

- 0=Not at all
- 1=Several days
- 2=More than half the days
- 3=Nearly every day
- Little interest or pleasure in doing things
- Feeling down, depressed, or hopeless

continue...

...Continuation

Table 1S. Self-administered questionnaire filled out by the participants

**GAD-2 Screening**
**In the past 2 weeks, how often have you been bothered by the following symptoms?**
• 0=Not at all
• 1=Several days
• 2=More than half the days
• 3=Nearly every day
• Feeling nervous, anxious, or on edge
• Unable to stop or control worrying
**Substance Use (Past 12 months)**
• Did you drink alcohol or use drugs more often or in larger amounts than intended? (0=No/1=Yes)
• Did you feel the need or desire to reduce alcohol or drug use? (0=No/1=Yes)
**Mental Health Services and Medications (Past 6 months)**
• Psychologist
• Psychiatrist
• Anxiolytics
• Antidepressants
• Antipsychotics
• None
**Household Income**
**Including your income and contributors to your support, what is the approximate monthly income per person in your household?**
• Less than BRL 1,000 per capita
• BRL 1,001–3,000
• BRL 3,001–5,000
• More than BRL 5,000
**Do you receive any financial aid to attend or maintain your studies?**
• Yes
• No
**If yes, what type of financial aid?**
• Scholarship
• Student loan
• Housing, transportation, or food support
• Other
**Do you work besides studying?**
• No
• Yes, full-time
• Yes, part-time
• Yes, occasionally

Table 2S. Linear regression model – Variables with p<0.15 in the univariate analysis

Variable	Reference	Beta	p value
Gender	Female	102.1	
	Male	-6.91	<0.001
	Non-binary	+10.5	0.11
Religion/Spirituality	No religion or spirituality	95.55	
	Practicing religion	+4.74	0.001
	Non-practicing religion	+4.69	0.002
	Spiritual, no religion	+5.26	<0.001
Clinical vs Surgical Preference	Extreme preference for surgery	92.75	
	Preference for surgery	+5.12	0.004
	Equal interest	+8.47	<0.001
	Preference for clinical	+8.15	<0.001
	Extreme preference for clinical	+10.08	<0.001
Frequency of Volunteering	Very frequent	101.67	
	Never	-4.41	0.034
	Rare	-2.64	0.199
	Occasional	-1.06	0.585
	Frequent	-1.55	0.481
Reason for Choosing Medicine	Interest in patient care	99.4	
	Academic/scientific interest	+0.1	0.954
	Humanitarian interest	+3.1	0.02
	Financial stability/monetary benefit	-4.1	0.091
	Scope of future professional practice	-0.6	0.747
	Only career option	-11.0	<0.001
	Social prestige + family imposition + no prior interest in medicine + other	-0.6	0.827
Self-perceived Empathy	Strongly agree	103.64	
	Partially agree	-7.28	<0.001
	Neutral	-15.11	<0.001
	Partially disagree	-16.39	<0.001
	Strongly disagree	-3.78	0.374
Number of People in Social Support Network	None	91.29	
	1–2	+6.64	0.159
	3–5	+8.28	0.069
	5–10	+9.01	0.049
	>10	+9.1	0.059
GAD-2 (Cut-off)	Below cut-off (0–2)	97.85	
	Above cut-off (3–6)	+4.68	<0.001
GAD-2 (Score Levels)	0	95.64	
	1	+4.74	0.003
	2	+4.83	0.002
	3	+5.16	0.005
	4	+7.2	<0.001
	5	+9.75	<0.001
	6	+12.02	<0.001
Use of Mental Health			
Any resources	None	98.29	
	Used	+2.58	0.009
Psychologist	Not used	98.74	
	Used	+2.3	0.024
Psychiatrist	Not used	99.17	
	Used	+1.96	0.103
Anxiolytics	Not used	99.06	
	Used	+2.71	0.03
Antidepressants	Not used	99.2	
	Used	+1.8	0.134
Antipsychotics	Not used	99.39	
	Used	+5.42	0.04

Table 3S. Multivariate regression model considering GAD-2 across all scores

Predictor	Estimate	Standard Error	p value
Gender			
Female	88.54	2.031	
Male	-5.89	0.947	<0.001
Non-binary	+5.62	6.317	0.374
Clinical vs. Surgical Preference			
Extreme preference for surgery	88.54	2.031	
Preference for surgery	+4.62	1.639	0.005
No preference/Equal interest	+6.86	1.743	<0.001
Preference for clinical	+7.02	1.617	<0.001
Extreme preference for clinical	+9.59	1.831	<0.001
GAD-2 Score			
0	88.54	2.031	
1	+4.34	1.484	0.004
2	+3.38	1.436	0.019
3	+4.17	1.716	0.015
4	+5.39	1.834	0.003
5	+8.07	2.278	<0.001
6	+10.75	1.8	<0.001
Spirituality/Religion			
Neither spiritual nor religious	88.54	2.031	
Practicing religious	+3.32	1.376	0.016
Non-practicing religious	+3.87	1.367	0.005
Spiritual but not religious	+3.18	1.437	0.027
